# Genetic and *in silico* functional characterization of a novel structural variant in the *PAH* gene by long-reads sequencing and structural modeling

**DOI:** 10.3389/fgene.2025.1669007

**Published:** 2025-09-17

**Authors:** Viviana Gallardo, Alexis Gaete, Jonathan Maldonado, Paulina Morales, Angela Peña, Valerie Hamilton, Víctor Faundes, Lorena Santa María

**Affiliations:** ^1^ Laboratory of Cytogenetics and Human Genomics, Institute of Nutrition and Food Technology, Universidad de Chile, Santiago, Chile; ^2^ Millennium Institute Center for Genome Regulation (CGR), Santiago, Chile; ^3^ Laboratory of Plant Multiomics and Bioinformatics, Department of Biology, Faculty of Chemistry and Biology, Universidad de Santiago de Chile, Santiago, Chile; ^4^ Millennium Institute of Integrative Biology (iBio), Santiago, Chile; ^5^ Laboratory of Inborn Errors of Metabolism, Institute of Nutrition and Food Technology, Universidad de Chile, Santiago, Chile

**Keywords:** phenylketonuria (PKU), *PAH* gene, CRISPR/Cas9, Oxford nanopore sequencing (ONT), exon duplication, protein modeling

## Abstract

**Introduction:**

Phenylketonuria (PKU) is an inherited metabolic disorder caused by biallelic variants in the *PAH* gene, leading to phenylalanine accumulation and progressive neuronal damage. Over 3,000 variants have been described worldwide; however, a previously unreported exon duplication was identified in Chile, whose genetic and functional characteristics remained unknown.

**Methods:**

A patient carrying a duplication of exon 2 in the *PAH* gene, previously detected by MLPA, was analyzed using nanopore sequencing coupled with CRISPR/Cas9 enrichment (nCATS) to determine the location, size, and orientation of the variant. Specific fragment amplification by PCR and Sanger sequencing was subsequently performed on samples from this patient and seven additional individuals to confirm the presence of the structural variant. Structural modelling of the resulting PAH protein was also conducted to predict functional consequences.

**Results:**

The nCATS technique identified a ∼18 kb tandem duplication between exons 1 and 3 of the *PAH* gene. This exon duplication was confirmed by PCR and Sanger sequencing in all eight patients. Additionally, an adenine insertion was detected at the junction site of the duplication. Structural modelling predicted an additional N-terminal segment that would likely interfere with sensing of phenylalanine.

**Discussion:**

The clinical, genetic and *in silico* functional characterization of this variant, using nCATS and structural modeling, suggests a mild, but relevant alteration in PAH enzymatic function. These findings support the delineation of genotype-phenotype correlations for complex structural variants, which may contribute to the development of personalized therapeutic strategies, while enriching both national and international PKU variant databases.

## 1 Introduction

Phenylketonuria (PKU) is an autosomal recessive inborn error of metabolism involving the amino acid phenylalanine (Phe). It is caused by biallelic variants in the gene encoding the enzyme phenylalanine hydroxylase (PAH), resulting in the toxic accumulation of Phe and its metabolites (Stone et al., 2021). The conversion of Phe to tyrosine (Tyr) requires both PAH activity and the cofactor tetrahydrobiopterin (BH_4_), whose regeneration is dependent on the enzyme dihydropteridine reductase (Campistol Plana, 2019). Defects in any of these components result in hyperphenylalaninemia (HPA), which can cause progressive neurological damage if left untreated (Stone et al., 2021; Williams et al., 2008).

The accumulation of Phe competitively inhibits the transport of large neutral amino acids into the brain, disrupting neurotransmitter synthesis and leading to neurological impairment (van Spronsen et al., 2017; van Wegberg et al., 2017). The degree of neurological involvement depends on plasma Phe concentrations, age at diagnosis, and treatment adherence (Ho and Christodoulou, 2014). Based on Phe levels at diagnosis and dietary tolerance, HPA is classified as classical PKU, moderate PKU, mild PKU, and mild HPA (Campistol et al., 2011).

Treatment should begin within the first month of life and consist of a Phe-restricted diet, avoiding foods rich in this amino acid and supplementing with Phe-free medical formulas to meet nutritional requirements (Stone et al., 2021). In Chile, since 1992, the National Newborn Screening Program for PKU and Congenital Hypothyroidism has enabled early diagnosis and treatment (Cornejo E., 2017). This dietary treatment is indicated for patients with Phe concentrations above 6 mg/dL and a Phe/Tyr ratio greater than 3.0 (Cornejo E. & Raimann B., 2004). Additionally, alternative therapies have been developed, including sapropterin (synthetic BH_4_) and phenylalanine ammonia lyase. Sapropterin is primarily effective in milder PKU phenotypes, where residual PAH activity is present, and provides limited or no therapeutic benefit for severe cases with complete loss of enzyme function (Nulmans et al., 2025; Somaraju and Merrin, 2015).

**FIGURE 1:**
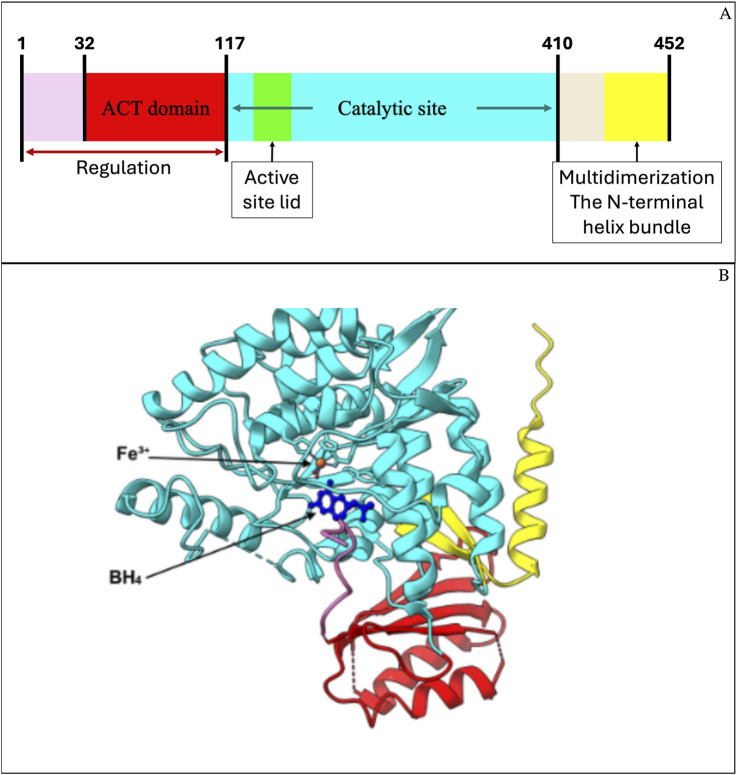
Structural domains of human phenylalanine hydroxylase (PAH) and predicted 3D model. **(A)** Schematic representation of PAH functional domains: the N-terminal regulatory region (residues 1–117), including the ACT domain (residues 32–117), the catalytic domain (residues 118–410), and the C-terminal multimerization domain (residues 411–452). **(B)** Predicted 3D model of PAH generated in UCSF ChimeraX using PDB structures 6N1K and 6HYC, which include iron (Fe^3+^) and the cofactor BH_4_. The ACT domain (red), catalytic domain (cyan), and C-terminal domain (yellow) are colored to match **(A)**. The mutation analyzed in this study is located within the N-terminal ACT domain, involved in allosteric regulation and phenylalanine binding.

PAH is a homotetrameric monooxygenase and consists of 452 amino acids organized into three structural domains: regulatory, catalytic, and oligomerization. The first one contains the ACT domain, which is required for sensing the levels of Phe and allowing the conformational change to an active state of the tetramer (Figure 1A) (Arturo et al., 2019). The catalytic domain contains the active site, where BH_4_ and Fe^3+^ coordinate with L-Phe to enable hydroxylation (Figure 1B). BH_4_ binding contributes to structural stabilization through a polar interaction network (Sanford and Keating, 2009).

The *PAH* gene is located on chromosome 12q23.2 and spans approximately 80–90 kb in the GRCh38 human genome assembly (NCBI Gene ID: 5053, accessed 2025; Liu et al., 2017). It comprises 15 exons and 14 introns, with exon 6 being the longest (∼197 bp) and exon 13 the shortest (∼44 bp), while intron 2 is the largest, spanning 17,874 bp (Cunningham et al., 2022). According to the BIOPKU database (accessed February 2025), over 3,450 variants of the *PAH* gene have been reported, with single nucleotide substitutions being the most prevalent type (∼80.5%) (Hillert et al., 2020). The gene has a GC content of 40.1%, which contributes to its high mutability (Vinogradov and Anatskaya, 2017).

The BIOPKU database currently compiles information from more than 23,000 patients across 51 countries, including genotypes, phenotypes, and BH_4_ responsiveness (BIOPKU, 2025). The PAHvdb locus-specific module reports 3,457 *PAH* gene variants as of February 2025. Globally, the most frequent variants are c.1222C>T (p.Arg408Trp), with an allele frequency (AF) of 22.2%, c.1066–11G>A (AF = 6.4%), and c.782G>A (p.Arg261Gln, AF = 5.5%). In Chile, in a study conducted by Hamilton et al. (2017) involving 71 patients diagnosed with PKU, the most prevalent variants identified were p.Val388Met (17.2%) and exon 5 deletion (14.9%). Biallelic variants were detected in 88.7% of cases, while in 11.3% only a single variant was found. In the latter group, MLPA (Multiplex Ligation-dependent Probe Amplification) analysis revealed a duplication of exon 2 (Hamilton et al., 2018; Stuppia et al., 2012), which had not been previously reported in the BIOPKU database or the scientific literature. However, the functional impact and clinical significance of this novel structural variant remain uncharacterized.

The aim of this study was to genetically characterize this exon 2 duplication in the *PAH* gene using CRISPR/Cas9-based targeted enrichment combined with long-read Nanopore sequencing. Additionally, we sought to predict its potential functional effect through *in silico* protein modeling using ChimeraX, and to analyze genotype–phenotype correlations based on plasma phenylalanine (Phe) levels in affected individuals as a proxy for enzymatic impact. Understanding the nature and functional consequences of this duplication may support more accurate pathogenicity classification, which is critical for genetic diagnosis and clinical decision-making. Characterizing this variant may help to clarify cases of HPA with unknown etiology and facilitate more effective risk stratification and personalized treatment strategies for Chilean patients with PKU.

## 2 Materials and methods

### 2.1 Clinical data and patient diagnosis

Eight patients were recruited in this study, in whom an exon 2 duplication was detected by MLPA analysis of the *PAH* gene (P055-D1 *PAH*, MRC-Holland^®^). They were diagnosed with PKU or HPA and are currently under clinical follow-up at the Institute of Nutrition and Food Technology (INTA). The diagnostic data revealed variability in phenylalanine (Phe) levels, ranging from 3.5 to 38 mg/dL (Supplementary Table S1). The Phe/tyrosine (Tyr) ratio was also elevated, varying from 0.58 to 10.45. The predominant clinical classification was “Mild” or “Moderate,” with one case classified as “Classical.” All patients and/or their legal guardians agreed to participate in this study by signing an informed consent form, approved by the local ethics committee.

Genetic characterization confirmed that all eight patients were compound heterozygotes carrying the exon 2 duplication in one allele. For three patients (PKU 1, PKU 2, and PKU 3), the second variant in *trans* was identified in a previous independent characterization study (see discussion section) (Hamilton et al., 2018). For the remaining five patients, only the exon 2 duplication has been confirmed by MLPA, while the identification of the second variant is part of an ongoing investigation whose results are not yet published.

Additionally, PCR-based confirmation of the exon 2 duplication was performed in all eight patients after its initial detection using Oxford Nanopore sequencing. The primers used for this confirmation and the amplification conditions are provided in Supplementary Table S3.

Twelve mL of peripheral blood was collected from all individuals carrying the duplication, and genomic DNA was extracted with the Wizard^®^ Genomic DNA Purification Kit (Promega, 2019), according to the manufacturer’s protocol.

### 2.2 Nanopore CRISPR/Cas9-targeted sequencing (nCATS)

#### 2.2.1 Targeted sequencing using CRISPR-Cas9 technology from Oxford nanopore technologies (ONT)

Targeted sequencing using CRISPR-Cas9 technology (Oxford Nanopore Technologies, ONT) was performed to obtain genomic information on the exon 2 duplication. The experiment was performed with DNA obtained from patient 8 (Supplementary Table S1). Synthetic CRISPR RNAs (crRNAs) and standardized trans-activating CRISPR RNAs (tracrRNAs) were purchased from Integrated DNA Technologies, Inc. (IDT, catalog #1072532). The crRNAs were designed using the IDT online tool, selecting those with the highest predicted on-target specificity and the lowest off-target potential. The selected crRNAs were CD.Cas9.FNBF0231.AD and Hs.Cas9.PAH.1.AB, as detailed in Supplementary Table S2 and Supplementary Figure S1.

Given that approximately 80% of duplications are tandem and in direct orientation (Newman et al., 2015), the crRNAs were designed to cleave complementary DNA strands flanking the region between exons 1 and 3 of the *PAH* gene. The reference sequence used to define this region was GenBank accession number AF404777.1. gRNA synthesis was performed according to the manufacturer’s protocol.

#### 2.2.2 Assembly of the ribonucleoprotein (RNP) complex

crRNAs and tracrRNAs were diluted in nuclease-free water to a final concentration of 100 μM each. The crRNAs and tracrRNAs were then combined to yield a final gRNA concentration of 10 μM. Ribonucleoprotein (RNP) complexes were assembled by mixing 10 μM of gRNA with 62 μM of HiFi Cas9 Nuclease V3 (IDT, 1081060) using the ONT Cas9 Sequencing Reaction Buffer Kit (SQK-CS9109), to a final volume of 100 μL. Each gRNA was prepared in a separate reaction. The mixtures were incubated at room temperature for 30 min and subsequently stored at 4 °C until use.

#### 2.2.3 Cas9 cleavage and library preparation

Genomic DNA from patient eight carrying the exon 2 duplication was dephosphorylated, cleaved by the respective RNP complexes, and ligated to sequencing adapters. Specifically, 3 μg of DNA were resuspended in 3 μL of 1X Reaction Buffer (SQK-CS9109) and dephosphorylated with 3 μL of phosphatase (SQK-CS9109) for 10 min at 37 °C, followed by a 2-min incubation at 80 °C. The sample was then maintained at room temperature, and 10 μL of the preassembled RNP complex (gRNA + Cas9) was added.

In the same tube, 1 μL of 10 mM dATP (SQK-CS9109) and 1 μL of Taq DNA Polymerase (SQK-CS9109) were added to perform A-tailing of the DNA ends. The sample was incubated at 37 °C for 20 min to allow RNP-mediated cleavage, followed by a 5-min incubation at 72 °C for A-tailing. Sequencing adapters were then ligated to the DNA ends using T4 DNA Ligase and ligation buffer (SQK-CS9109) for 10 min at room temperature.

The resulting DNA was purified using 0.3X AMPure XP Beads (Beckman Coulter, A63881), washed twice on a magnetic rack with Long Fragment Buffer (SQK-CS9109), and eluted in 15 μL of Elution Buffer (SQK-CS9109), thus generating the final DNA library. Finally, 12 μL of the DNA library were combined with 37.5 μL of Sequencing Buffer (SQB, SQK-CS9109) and 25.5 μL of Loading Beads (SQK-CS9109), making the sample ready for sequencing.

#### 2.2.4 Nanopore sequencing

The libraries obtained from the previous step were loaded onto a MinION flow cell (MIN-101B) and sequenced using a Mk1B MinION device (Oxford Nanopore Technologies). Raw signal data generated in fast5 format were basecalled into nucleotide sequences (fastq format) using Guppy software version 6.2.1 (Oxford Nanopore Technologies) (Wick et al., 2019). Adapter sequences were subsequently trimmed using Porechop version 0.2.4 (https://github.com/rrwick/Porechop) (Wick et al., 2017). Read quality was assessed using longQC.py version 1.2.0 (Fukasawa et al., 2020) and NanoPlot version 1.2.0 (De Coster et al., 2018). After visual inspection of the quality distribution and read length (Supplementary Figure S2), reads with a minimum quality score >Q10 and length ≥500 bp were selected using NanoFilt version 2.8.0 (De Coster et al., 2018).

Filtered reads were aligned by homology (mapping) against a segment of chromosome 12 (*Homo sapiens*), corresponding to the *PAH* transcript region extended by 10,000 bases upstream and downstream, which also encompasses the region used to design the gRNAs. This target region spans nucleotides 102,826,651–102,968,295 of chromosome 12 in the GRCh38.p13 human genome assembly, totaling 141,644 bases. The *Homo sapiens* genomic sequence used for mapping was downloaded from Ensembl (Cunningham et al., 2022), and alignment was performed using NGLMR version 0.2.7 (Sedlazeck et al., 2018).

After mapping, structural variant (SV) detection was performed using Sniffles version 2.0.7 (Sedlazeck et al., 2018) and NanoVar version 1.4.1 (Tham et al., 2020), with a minimum of 10 supporting reads required to define the precise breakpoint of each variant. Reads containing relevant SVs were subsequently assembled using Wtdbg2 (Ruan and Li, 2020) and CLC Genomics Workbench version 12 (QIAGEN Aarhus, 2015), following each software’s guidelines.

### 2.3 Variant confirmation testing

The following procedures were performed in all patients in whom the exon 2 duplication was initially detected by MLPA, including the patient analyzed by the nCATS technique.

#### 2.3.1 Junction site amplification by PCR

To confirm the location of the duplication in the newly identified PAH gene variant, a primer pair was designed (Supplementary Table S3) using the Primer-BLAST tool (https://www.ncbi.nlm.nih.gov/tools/primer-blast/). These primers flank a specific sequence that spans the junction between the original region and the adjacent duplication, which is only present in the allele carrying the novel *PAH* variant.

To perform the PCR reaction, FastStart Taq DNA Polymerase was used (F. Hoffmann-La Roche Ltd., Basel, Switzerland), which provides high yield for sequences with high GC content, such as the exon 1 region of the *PAH* gene. GC-rich regions are known to form stable secondary structures and exhibit high melting temperatures, which hinder amplification using standard DNA polymerases. The selection of a high-fidelity, GC-optimized polymerase was therefore crucial for ensuring efficient amplification of this target region (Naz and Fatima, 2013). The manufacturer’s protocol was adjusted to use a final concentration of Taq polymerase of 2U, PCR buffer 10X to 1X, MgCl_2_ at 0.5 mM, dNTPs at 10 μM, 0.2 μM of each primer and 100 ng of DN, in a final reaction volume of 25 μL. Amplification was carried out under the following conditions: an initial denaturation cycle at 94 °C, followed by 35 cycles of denaturation, primer annealing, and extension at 94 °C, 58 °C, and 72 °C, respectively, and a final extension at 72 °C. The reaction was then held at 4 °C until further use.

#### 2.3.2 Verification fragment sequencing

To determine the exact nucleotide sequence at the duplication junction in the affected patients, the previously amplified fragments were subjected to Sanger sequencing (Singh et al., 2022).

The PCR product was purified using silica columns and the PureLink™ PCR Purification Kit (Invitrogen, K310001) according to the manufacturer’s instructions.

The fragments were sequenced using the BigDye™ Terminator v3.1 Cycle Sequencing Kit (Applied Biosystems, ThermoFisher Scientific, 4337455). The mixture was subjected to 25 thermal cycles, beginning with an initial denaturation at 96 °C, followed by primer annealing at a temperature determined by the primers’ Tm, and extension at 60 °C for a time adjusted to the fragment length.

A volume of 25–30 μL of the purified products were loaded into the SeqStudio Genetic Analyzer (Applied Biosystems) and sequenced using the manufacturer’s recommended parameters. The results were analyzed using Sequencing Analysis Software version 7. Basecalling data and electropherograms were obtained for each sample.

#### 2.3.3 Protein model prediction based on the altered gene sequence

The crystal structure of human PAH solved by X-ray diffraction at a resolution of 1.67 Å (PDB entry: 6n1k) was used as the reference, as it represents the most complete structural model of human PAH to date (Figure 1B) (Arturo et al., 2019). However, several regions could not be reliably crystallized, and these vary depending on the monomer considered. One consistently unresolved region spans amino acids 1–18. Although exon 2 encodes residues 21–56, it was necessary to complete the missing N-terminal region (residues 1–18) as well as any additional segments potentially affected by the introduction of the duplicated exon 2.

In this context, *in silico* modeling of the missing PAH regions was performed using UCSF ChimeraX software (Meng et al., 2023). Chain C of the crystallized PAH tetramer was used as a template, as it contains the fewest unresolved regions and includes all residues encoded by exon 2 crystallized with high confidence, except for residue #21. The missing segments were modeled using the “Model Loops” tool in ChimeraX, generating five predicted loop conformations based on the DOPE-HR protocol. The final model was selected based on the best structural prediction scores: a GA341 score close to or equal to 1 and a more negative zDOPE score. The completed PAH model was then used to generate a variant model incorporating the residues encoded by the duplicated exon 2.

To generate a PAH protein model containing the duplicated amino acid segment encoded by exon 2, a FASTA file was created with the corresponding modified PAH sequence. Using this sequence and the previously completed full PAH model as a template, five structural models incorporating the duplication were generated using the “Modeller Comparative” tool. The model with the best GA341 and zDOPE scores, as described previously, was selected.

## 3 Results

### 3.1 Detection of structural variants by nanopore sequencing

Quality value (QV)-based filtering resulted in less than 10% data loss in both sequencing directions. Sequenced fragments reached lengths exceeding 109 kb (Supplementary Table S4; Supplementary Figure S1). A total of 27,587 aligned reads were obtained, including 11,197 reads in the 5′ to 3′ direction and 16,390 in the 3′ to 5′ direction, spanning the genomic region between exons 1 and 3 of the *PAH* gene in the PKU patient harboring the structural variant of interest.

**FIGURE 2:**
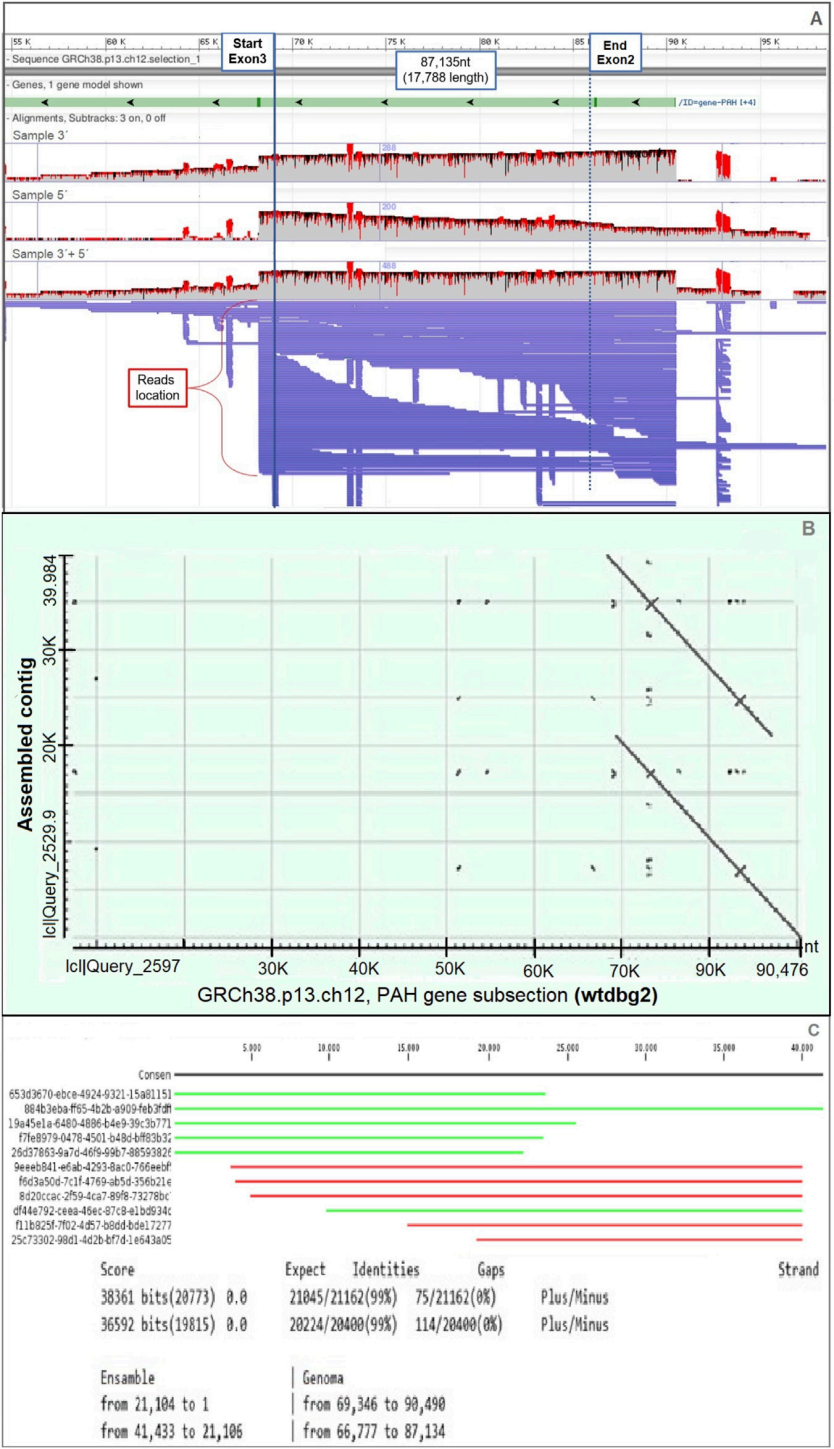
Evidence of tandem duplication of exon 2 in the *PAH* gene. **(A)** Long-read Nanopore sequencing data aligned to the *PAH* gene region (GRCh38.p13, chromosome 12), visualized using Sniffles. Continuous long reads span from exon 3 to a repeated exon 2 region, suggesting a tandem duplication. **(B)** Dot plot of the assembled contig using the *wtdbg2* assembler, showing repeated segments aligned across the exon 2 genomic region. **(C)** Alignment results of the duplicated sequence against the reference database, performed using CLC Genomics Workbench. Partial alignments (in green and red) highlight the presence of duplicated and rearranged sequences mapped on both DNA strands.

Tandem duplication of exon 2 was predicted using Sniffles v2.0.7 by mapping long-read data to the human reference genome (Figure 2A). The duplication spans 17,788 bp and was supported by 14 long reads. After sequence assembly, a second “double-match” event at the same genomic location confirmed the presence of the duplication (Figure 2B). Furthermore, we were able to determine that 11 out of the 14 reads with duplication signals showed structural consistency upon alignment with the assembled contigs, reinforcing the duplication evidence (Figure 2C).

The analysis of nanopore sequencing data using three independent bioinformatic tools confirmed the presence of a 17,788 bp tandem duplication in the *PAH* gene, as illustrated in the schematic representation in Figure 2A. This approach enabled the precise determination of the genomic coordinates of the duplicated segment, spanning from position 102,913,786 to 102,895,995 (reverse strand) based on the GRCh38.p13 human genome assembly.

### 3.2 Validation of the tandem duplication by PCR in HPA and PKU patients

**FIGURE 3:**
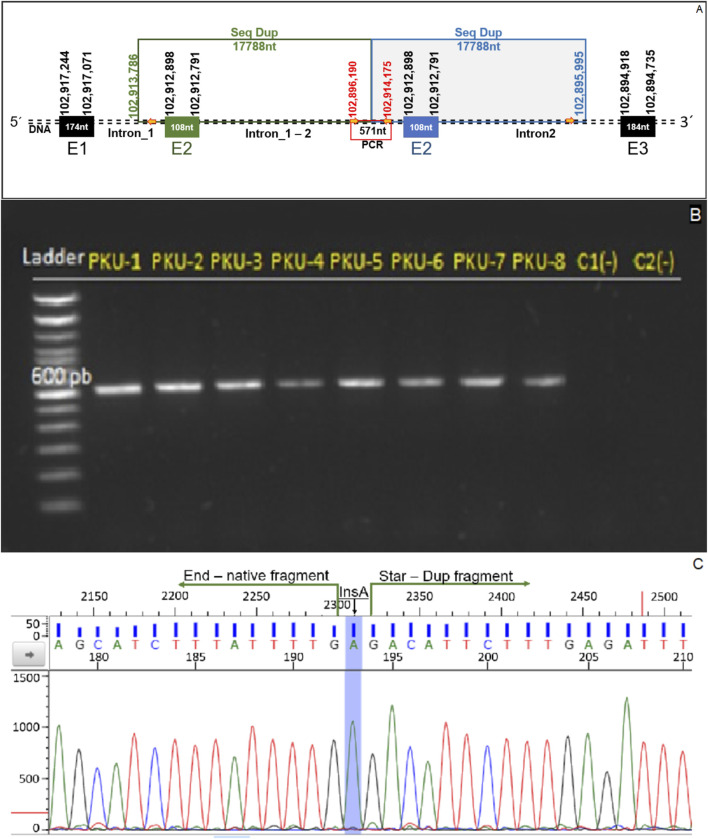
Genomic confirmation of tandem exon 2 duplication. **(A)** Schematic representation of the duplication detected in the *PAH* gene, showing the repeated 17,788-nucleotide segment encompassing exon 2 and parts of adjacent intronic regions. The primers used for PCR amplification are indicated by red arrows, and the expected amplicon of 571 bp spans the junction of the duplicated segment. **(B)** Agarose gel electrophoresis of PCR products from patients PKU-1 to PKU-8, showing a distinct 571 bp band indicative of the tandem duplication. Two control samples (C1 and C2) were included and showed no amplification. **(C)** Sanger sequencing chromatogram confirming the breakpoint junction between the native and duplicated fragments. An adenine insertion (InsA) was detected at the fusion point, marked in blue.

The primers were designed to flank the junction region of the duplicated segment. These primers flank an approximately 600 bp DNA fragment located at the junction between the original and duplicated segments (Figure 3A; Supplementary Table S3).

Genomic DNA from eight PKU patients carrying the suspected duplication and two healthy controls was included in the assay. All patients tested positive, as evidenced by amplification of the duplication junction, while no amplification was observed in the control samples (Figure 3B).

Sanger sequencing of the amplified fragment flanking the duplication junction enabled the determination of the exact genomic position of the duplicated segment and whether the insertion site was consistent across all patients. The sequences obtained from the eight individuals carrying the duplication were aligned against the human genome reference (GRCh38.p13). The mapping confirmed the presence of the tandem duplication with identical physical coordinates in all patients studied. Additionally, all sequenced individuals exhibited an adenine (A) insertion precisely at the junction site of the tandem duplication (Figure 3C).

Based on these sequencing results and following the most recent guidelines of the International System for Human Cytogenomic Nomenclature (ISCN, 2024), the genomic description of the variant is as follows:

− Seq [GRCh38]dup (12)(12q23.2q23.2)ins (12)(12q23.2q23.2)
− NC_000012.12:g.102895995_102913786dup [NC_000012.12:g.102895994insA]

### 3.3 *In silico* protein modeling and functional impact prediction

The detected duplication encompasses the entire exon 2. Based on the sequences of exons 1 and 3 and the tandem orientation of the duplicated segment, this variant is not expected to induce degradation of the mature transcript, as it does not alter the reading frame. Furthermore, the biochemical profile observed in patients carrying the duplication was consistent even with hyperphenylalaninemia (HPA). This suggests that the elevated Phe levels are primarily attributable to the pathogenic variant located on the other allele, while the residual PAH retains partial functionality.

**FIGURE 4:**
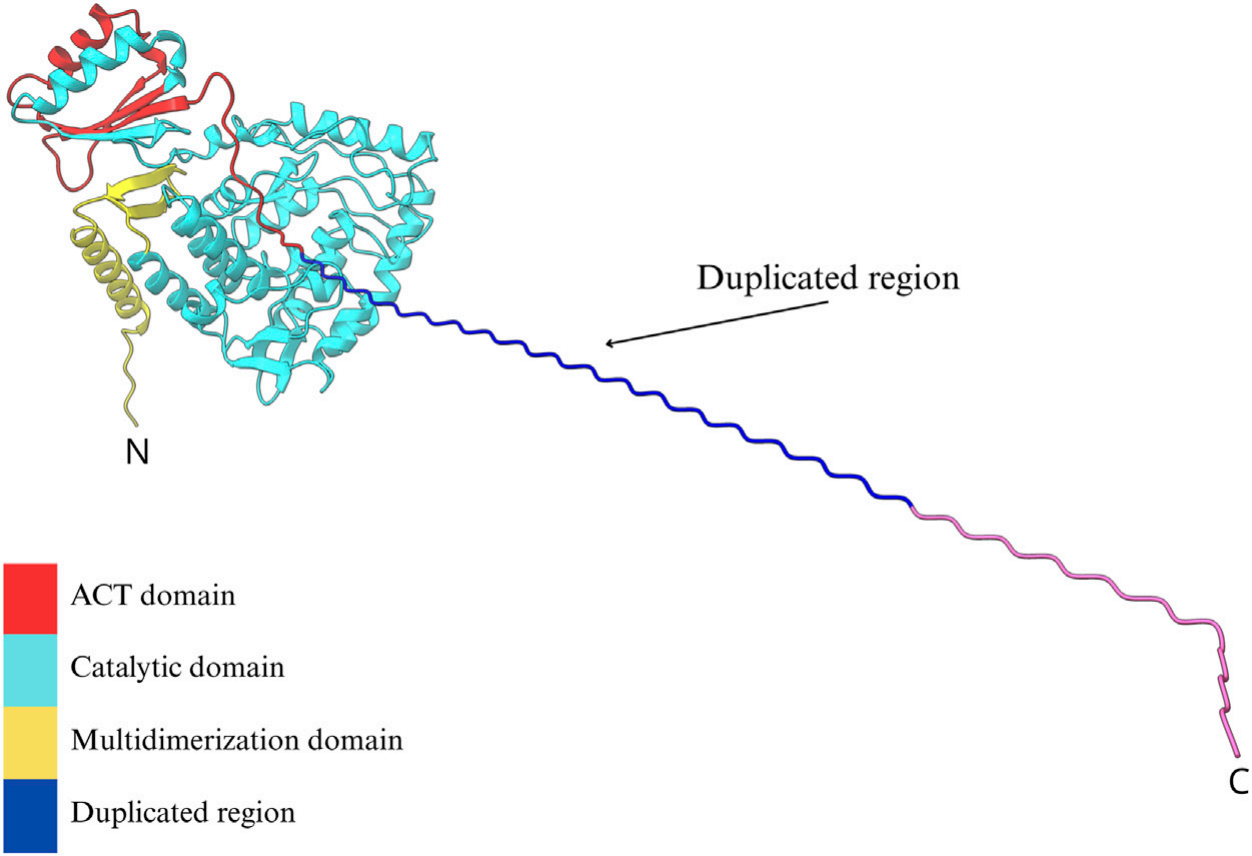
*In silico* predicted tridimensional model of PAH harboring a tandem duplication of residues encoded by exon 2. The canonical PAH structure is shown with its functional domains: the regulatory ACT domain (red), the catalytic domain (cyan), and the tetramerization domain (yellow). The duplicated region, inserted at the N-terminal portion of the protein, is visualized as an extended helical structure (blue to magenta gradient) projecting outward from the native fold. This alteration could potentially affect the ACT domain and the conformational change of the tetramer. The model was generated using a homology-based approach in ChimeraX.

To investigate the potential structural impact of the duplicated exon 2, a three-dimensional model of the PAH protein was generated using the *in silico* tandem duplication and refined with the “Model Loops” tool in ChimeraX. The predicted model revealed an elongated N-terminal extension, without a defined secondary structure, adjacent to the native exon 2 sequence. Despite this addition, the overall architecture of the protein was largely preserved compared to the wild-type model. Minor deviations were noted in the autoregulatory domain, indicating local conformational changes (Figure 4).

## 4 Discussion

In 2017, a national genetic landscape of variants present in Chilean patients with Phenylketonuria (PKU) was established, expanding the molecular epidemiology of the disease-causing mutations. Among the findings, an uncharacterized genomic duplication was identified. This structural alteration raised essential questions regarding its precise genomic location and potential impact on PAH function. These uncertainties were addressed through recent technological advances in long-read sequencing.

Given that PKU/HPA is an autosomal recessive disorder, in which both alleles must be affected to cause a pathological increase in Phe, and one of the alleles in these patients is fully deleterious (manuscript in review), that is, it has a variant that is associated with a complete lack or significant reduction of the PAH catalytic activity and a classical PKU phenotype (see published variants below)-the residual PAH must be present and yet functionally deficient. Of note, that MLPA was prioritized for the study of these patients as exon deletions/duplications explain ∼17% of alleles with pathogenic variants in Chilean patients with PKU (Hamilton et al., 2018). On the other hand, as MLPA has a low cost and it is a straightforward technique, it is more practical and efficient to start with this technique in Chilean patients with PKU. Once an exon deletion/duplication is detected, Sanger sequencing of the commonest variants are performed (Hamilton et al., 2018), which has allowed us to reduce the price of genetic testing in these patients.

Importantly, the duplicated exon 2 lies within the N-terminal region of the protein, which is unlikely to significantly disrupt the catalytic site (Figure 4). Moreover, as PAH functions as a tetramer that requires a conformational change to be active, the presence of an additional N-terminal segment may interfere with proper sensing of Phe by ACT domain and the subsequent conformational change to an active state of the PAH tetramer (Arturo et al., 2019). However, we cannot discard that this additional segment may affect tetramer assembly and subsequently compromise the enzymatic function. Both considerations support the hypothesis that the duplication leads to a moderately reduced enzymatic activity, consistent with the mild biochemical phenotype observed in some patients carrying this variant.

The duplication of exon 2 in *PAH* was precisely characterized through CRISPR/Cas9-mediated genome excision, which enabled targeted sequencing of the region of interest. Additionally, Oxford Nanopore Technologies (ONT) long-read sequencing allowed the generation of high-resolution reads spanning the entire duplicated segment and its configuration. This combination of techniques was essential for a comprehensive analysis of the genetic variant, providing accurate data of both size and genomic position. Subsequent verification using PCR and sequencing of the junction site confirmed the presence and structure of this *PAH* variant in all carriers. These findings pave the way for a cost-effective and straightforward molecular screening method in future PKU or HPA patients to detect potential exon 2 duplications.

Fragment duplications can result from genetic recombination processes involving the exchange of DNA sequences between two similar or identical molecules. This mechanism fulfills several biological functions, such as double-strand break repair, the generation of genetic diversity during meiosis, and horizontal gene transfer (Magadum et al., 2013). Analysis of nanopore sequencing data revealed a large genomic fragment duplication near a 305 bp Alu element located within the *PAH* gene (Figure 3A) (Haeussler et al., 2019). A distinguishing feature of homologous recombination is its requirement of high sequence similarity between the regions undergoing recombination. One of the primary sources of such homologous sequences is Alu elements repetitive sequences scattered throughout the human genome that can mediate homologous recombination events (Stenger et al., 2001). Homologous recombination between Alu elements is known to cause chromosomal rearrangements, including duplications (Cordaux and Batzer, 2009), which provides a plausible mechanism for the structural variant identified in the studied patients and supports its pathogenic classification.

An important aspect to address is the phenotype observed in the studied patients. The genotype–phenotype classification follows Guldberg’s prediction system, which states that the clinical phenotype of PKU patients depends on the milder of the two alleles (Guldberg et al., 1998). When analyzing patients carrying the exon 2 duplication, the other allelic variants identified in the study by Hamilton et al. (2018a) would predict a classical PKU phenotype, based on data reported in the BIOPKU database. This classical form typically requires strict dietary restriction of phenylalanine and represents a greater clinical challenge. However, the biochemical phenotype observed in these patients, based on plasma Phe and Tyr levels, was either mild or moderate. This suggests that the duplicated allele may encode a partially functional enzyme with residual activity sufficient to metabolize phenylalanine to some extent. This interpretation is further supported by clinical observations in our cohort, where three patients carrying both the exon 2 duplication and a second allelic variant previously associated with a classical phenotype (c.728G>A, p.Arg243Gln; c.782G>A, p.Arg261Gln; c.1162G>A, p.Val388Met) consistently presented with milder biochemical profiles (mild or moderate PKU). Family analyses were performed in these three initial patients, confirming the presence of the exon 2 duplication in one of the corresponding parents through MLPA (data from an independent study currently under review). In future, the PCR-based confirmation assay developed here could be applied to first-degree relatives to validate the inheritance pattern in an easier and cheaper way.

According to the BIOPKU database, these three missense variants affect highly conserved amino acids in the PAH enzyme, impairing its folding, structural stability, and/or catalytic activity. As a result, they are associated with significantly reduced enzymatic activity and the manifestation of classical phenylketonuria (BIOPKU, 2025). These findings suggest that the duplicated allele may retain partial enzymatic activity, contributing to a less severe clinical presentation than expected. Accordingly, the structural variant is consistent with a mild to moderate phenotype, indicating that affected individuals may benefit from a moderately restricted diet and/or pharmacological treatment to enhance phenylalanine tolerance.

Another finding derived from the sequencing of the genetic alteration was the insertion of an adenine at the junction of the duplicated fragments, as shown in Figure 3C. The human reference genome serves as a genomic backbone for sequence alignment and the identification of genomic variations (Hehir-Kwa et al., 2016). However, a complete set of human reference genomes capable of capturing the full spectrum of population-level genetic variation, namely, a pangenome, has not yet been established (Taylor et al., 2025). Although the reference genome assembly used in this study (GRCh38.p13) represents the penultimate version and includes only 261 alternative loci to account for haplotype diversity (Schneider et al., 2017), to date, no Latin American-specific reference genome is available that would provide a representative sequence for the patient studied. This context may help explain the presence of the adenine insertion, which was consistently observed in all patients analyzed.

Although the exon 2 tandem duplication was estimated not to disrupt the reading frame of the transcript since the number of duplicated nucleotides is a multiple of three, thus preserving codon phase (Cooper, 2019) the prediction of its folding proved neither accurate nor conclusive. This can be explained, firstly, because at the time of this study, available bioinformatic tools did not allow precise *de novo* modeling of events such as duplications or deletions of amino acid segments; instead, they relied on structural predictions based solely on homology with previously characterized proteins. Secondly, the whole three-dimensional structure of the PAH protein has not yet been fully resolved, particularly in the N-terminal region encompassing the first 18 amino acids (mostly encoded by exon 1), which limits the ability to predict the conformational consequences of the duplication in that region.

Given these limitations, it is necessary to investigate this variant using functional characterization models that can provide direct evidence of its biochemical and enzymatic behavior. Thus, further studies using cellular models should be planned, both *in vitro*, employing immortalized human hepatocyte lines such as HepaRG, derived from hepatocellular carcinoma and widely used in metabolism research (Guillouzo et al., 2007) and *ex vivo*, through the generation of hepatocytes from fibroblasts of affected patients using reprogramming with induced pluripotent stem cells (iPSC) (Garcia-Llorens et al., 2023). These models would enable the isolation of the mutant PAH, determination of its molecular weight, assessment of its catalytic activity, and potentially the resolution of its three-dimensional structure by crystallographic methods, ultimately aiming to elucidate the functional impact of this duplication in the clinical context of PKU and HPA.

While dietary phenylalanine restriction remains the first-line treatment for PKU, sapropterin, a synthetic form of the BH_4_ cofactor that enhances residual PAH activity, has been shown to reduce plasma Phe levels in responsive patients (Wiedemann et al., 2020). The mechanisms that determine responsiveness to sapropterin are multifactorial and complex, making it difficult to establish a direct correlation between genotype and drug response (Muntau et al., 2019). Upon analyzing the three-dimensional model of PAH generated through the *in silico* prediction of the genomic variant, we observed that the structural alteration caused by the exon 2 duplication affects the N-terminal autoregulatory tail of PAH, where the BH_4_ cofactor binding site is, and potentially a region involved in the sapropterin mechanism of action (Arturo et al., 2019). Based on this and considering the disruption of this region by the duplication, we could hypothesize that patients carrying this variant may not respond to the drug. However, as previously mentioned, the mechanisms of action of sapropterin dihydrochloride are multifactorial, underscoring the need to expand studies to determine the responsiveness of these patients and to further characterize this variant for optimizing PKU treatment.

Despite the aforementioned challenges, this study demonstrates that the genetic characterization of variants in PKU patients enables *in silico* estimation of protein functionality and provides a framework for future studies to confirm these predictions *in vitro* or *ex vivo*. Moreover, these analyses allow the prediction of clinical phenotype and potential treatment responsiveness. This is particularly relevant in PKU, where partial loss of protein function may allow the use of cofactors to enhance enzymatic activity, potentially avoiding the need for a highly restrictive and consequences of variable adherence to diet.

## Data Availability

The datasets presented in this study can be found in online repositories. The names of the repository/repositories and accession number(s) can be found below: https://www.ncbi.nlm.nih.gov/, PRJNA1288462.
