# Supplementary material for: Genetic and in silico functional characterization of a novel structural variant in the PAH gene by long-reads sequencing and structural modeling
Source: Front Genet. 2025 Sep 17;16:1669007. doi: 10.3389/fgene.2025.1669007 (PMC12483855; doi:10.3389/fgene.2025.1669007)
Supplement: Supplementary file 1 [file DataSheet1.docx]

Supplementary Material

**Index Supplementary Tables**

[Supplementary Table 1. Biochemical phenotype at diagnosis for patients with PAH gene variants. 2](#_Toc202915588)

[Supplementary Table 2. crRNA Design Data 2](#_Toc202915589)

[Supplementary Table 3. Primer sequences used for the confirmation PCR test 3](#_Toc202915590)

[Supplementary Table 4. Read data obtained through Nanopore sequencing 3](#_Toc202915591)

**Index Supplementary Figures**

[Supplementary Figure 1. Design and selection of CRISPR RNAs (crRNAs) targeting the PAH gene for Cas9-based enrichment 4](#_Toc202915275)

[Supplementary Figure 2. Quality and length distribution of nanopore sequencing reads 5](#_Toc202915276)

# Supplementary Tables

Supplementary Table 1. Biochemical phenotype at diagnosis for patients with PAH gene variants.

| Sample Code | Diagnostic Phe (mg/dL) | Diagnostic Tyr (mg/dL) | Phe/Tyr | Classification |
| --- | --- | --- | --- | --- |
| PKU 1 | 4,4 | 0,63 | 6,98 | Mild |
| PKU 2 | 3,5 | 6 | 0,58 | Mild HPA |
| PKU 3 | 15,6 | 2,49 | 6,27 | Moderate |
| PKU 4 | 10,2 | 1,6 | 6,38 | Mild |
| PKU 5 | 10 | 2,6 | 3,85 | Mild |
| PKU 6 | 38 | n/a | n/a | Classic |
| PKU 7 | 11,5 | 1,1 | 10,45 | Moderate |
| PKU 8 | 11,9 | 1,2 | 9,92 | Moderate |

Phenylalanine (Phe) and tyrosine (Tyr) concentrations (mg/dL) measured at diagnosis were used to calculate the Phe/Tyr ratio and classify the phenotype of each patient. The classification was based on international guidelines. **n/a**: no information available.

Supplementary Table 2. crRNA Design Data

| Design ID | CD.Cas9.FNBF0231.AD | Hs.Cas9.PAH.1.AB |
| --- | --- | --- |
| Gene Symbol | PAH | PAH |
| Position | 84 | 77 |
| Strand | - | + |
| Sequence | AGCCAGAGACCTCACTCCCG | GCAGGCTACGTTTATCCAAA |
| PAM | GGG | TGG |
| On-Target Score | 62 | 60 |
| Off-Target Score | 58 | 78 |
| SNP Location | N/A | N/A |

crRNA design data for CRISPR/Cas9 targeting of the *PAH* gene. Includes sequence, strand orientation, PAM site, and on- and off-target scores for each design.

Supplementary Table 3. Primer sequences used for the confirmation PCR test

| Oligo Name | Primer Type | Sequence (5'-3') | Tm (°C) | GC% |
| --- | --- | --- | --- | --- |
| DupE2F_1 | Forward | TCTTGCCATCCCAATGAGGC | 60.40 | 55.00 |
| DupE2R_1 | Reverse | GGAACATGCACAGGCTTTTGA | 59.66 | 47.62 |

Forward and reverse oligonucleotides are listed along with melting temperatures and GC content.

Supplementary Table 4. Read data obtained through Nanopore sequencing

| Read Direction | SEQ 5′–3′ | SEQ 3′–5′ |
| --- | --- | --- |
| Number of reads | 89,733 | 147,195 |
| Total bases | 867.7 Mb | 1328 Mb |
| Maximum read length | >109 Kb |  |
| Q10 and 500 bp fragments (after filtering) | SEQ 5′–3′ | SEQ 3′–5′ |
| Number of reads | 83,646 (93.2%) | 142,450 (96.8%) |
| Total bases | 807.5 Mb (44X coverage) | 1284.6 Mb (71X coverage) |

Summary of read metrics from Nanopore sequencing used to detect the *PAH* exon 2 tandem duplication. Includes direction of reads, total bases, maximum read length, and quality-filtered data.

## Supplementary Figures


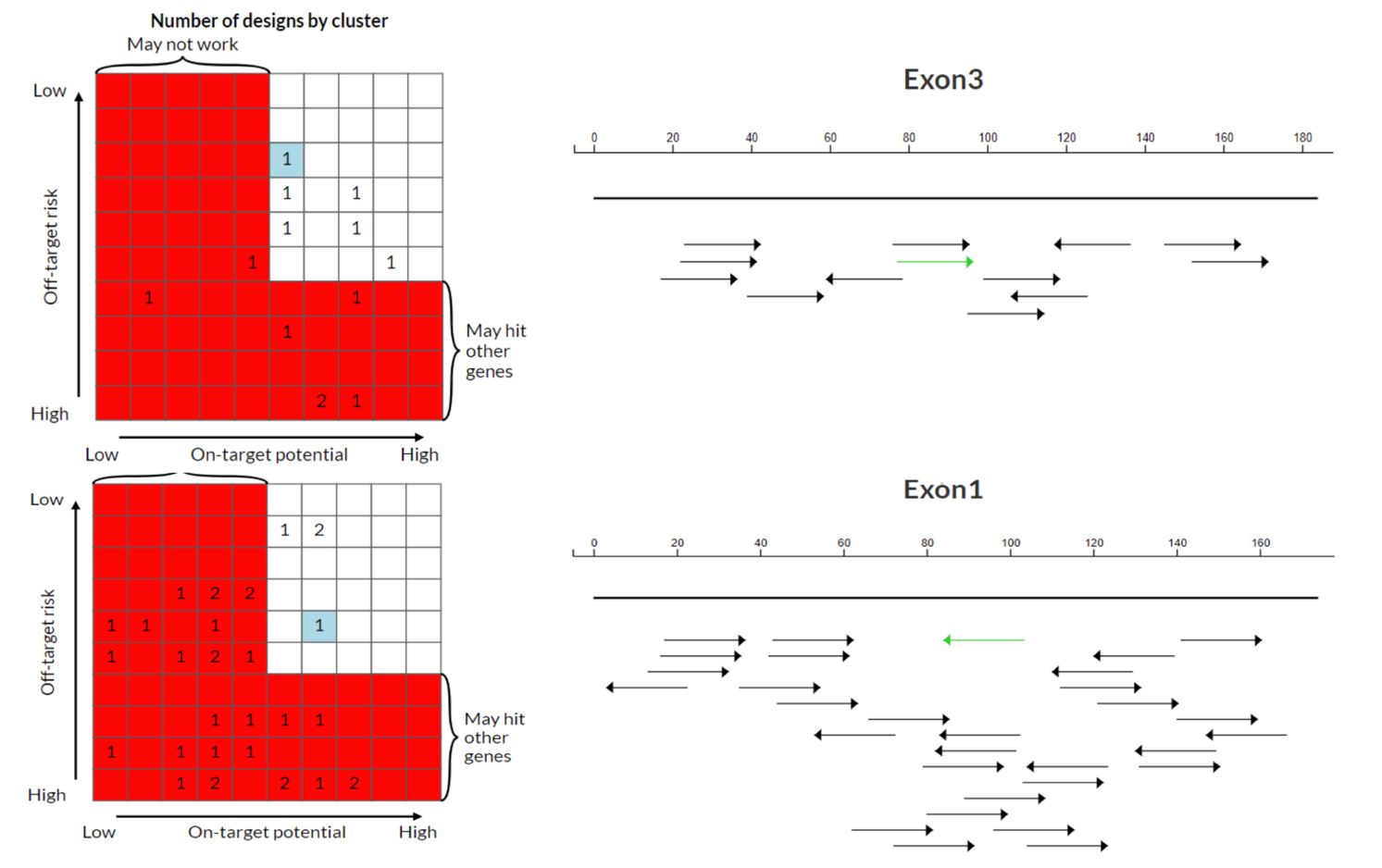


Supplementary Figure 1. Design and selection of CRISPR RNAs (crRNAs) targeting the PAH gene for Cas9-based enrichment

The diagrams show the on-target potential and off-target risk scores (left) and target locations on the PAH gene (right) for exon 1 and exon 3. Green arrows indicate the crRNAs selected for Cas9 cleavage: CD.Cas9.FNBF0231.AD and Hs.Cas9.PAH.1.AB. See Table S2 for sequence details.


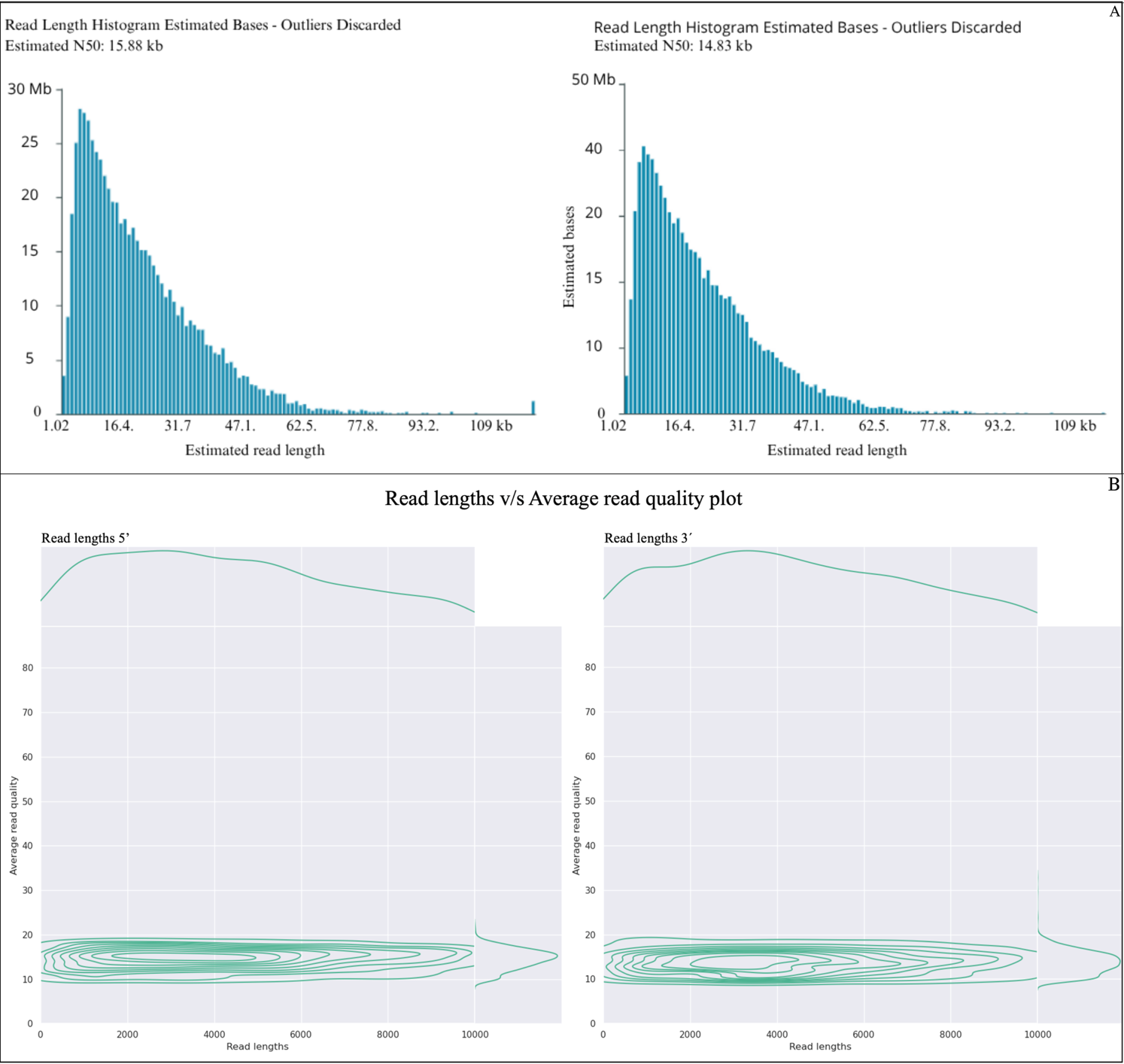


Supplementary Figure 2. Quality and length distribution of nanopore sequencing reads

(A) Histograms showing estimated read length distributions for two samples after outlier removal. The N50 values were 15.88 kb and 14.83 kb, respectively.

(B) Contour plots depicting the relationship between read length and average quality score for 5′ and 3′ ends. Most reads showed consistent quality around Q10 across lengths, with slight variations at the extremities.
